# Submesoscale ocean fronts act as biological hotspot for southern elephant seal

**DOI:** 10.1038/s41598-019-42117-w

**Published:** 2019-04-03

**Authors:** Lia Siegelman, Malcolm O’Toole, Mar Flexas, Pascal Rivière, Patrice Klein

**Affiliations:** 1 Univ. Brest, CNRS, IRD, Ifremer, LEMAR, Plouzané, France; 2 California Institute of Technology, Pasadena, CA USA; 3 Jet Propulsion Laboratory, California Institute of Technology, Pasadena, CA USA; 4 UWA Oceans Institute, Indian Ocean Marine Research Centre, University of Western Australia, Crawley, WA 6009 Australia

## Abstract

The area west of the Kerguelen Islands (20–70°E/45–60°S) is characterized by a weak mesoscale activity except for a standing meander region of the Antarctic Circumpolar Current (ACC) localized between 20 and 40°E. A unique bio-physical dataset at high-resolution collected by a southern elephant seal (*Mirounga leonina*) reveals a conspicuous increase in foraging activity at the standing meander site up to 5 times larger than during the rest of her three-month trip west of the Kerguelen Islands. Here, we propose a physical explanation for such high biological activity based on the study of small-scale fronts with scales of 5 to 20 km, also called submesoscales. The standing meander is associated with intensified frontal dynamics at submesoscale, not observed in the rest of the region. Results shed new light on the spatial distribution of submesoscale fronts in the under-sampled area west of the Kerguelen plateau and emphasize their importance for upper trophic levels. Despite that most elephant seals target foraging grounds east of the Kerguelen Plateau, our findings suggest that excursions to the west are not accidental, and may be explained by the recurrently elevated physical and biological activity of the site. As such, other standing meanders of the ACC may also act as biological hotspots where trophic interactions are stimulated by submesoscale turbulence.

## Introduction

**Figure 1:**
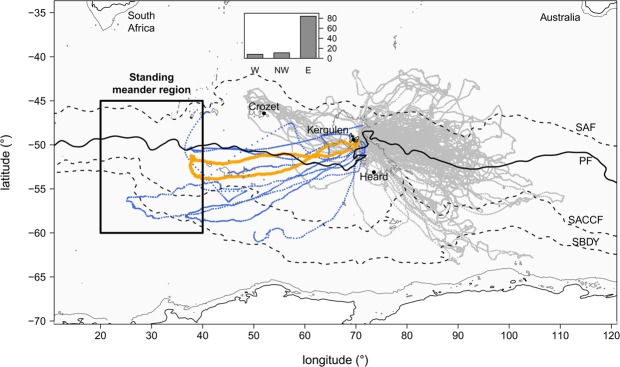
Spatial distribution of the 103 post-breeding female southern elephant seals tagged from the Kerguelen Islands since the beginning of the Marine Mammal Exploring the Ocean Pole-to-Pole consortium in 2004. Southern elephant seals are separated per region: west (W) in blue and east (E) and northwest (NW) in gray. Insert figure shows regional seal distribution. The high-resolution trajectory considered in this study is shown in orange. The region of the standing meander is identified by the black rectangle^2^. Climatological position of the Sub-Antarctic Front (SAF), Polar Front (PF), Southern Antarctic Circumpolar Current Front (SACCF) and Southern Boundary front (SBDY) are indicated in black according to Kim and Orsi^70^.

**Figure 2:**
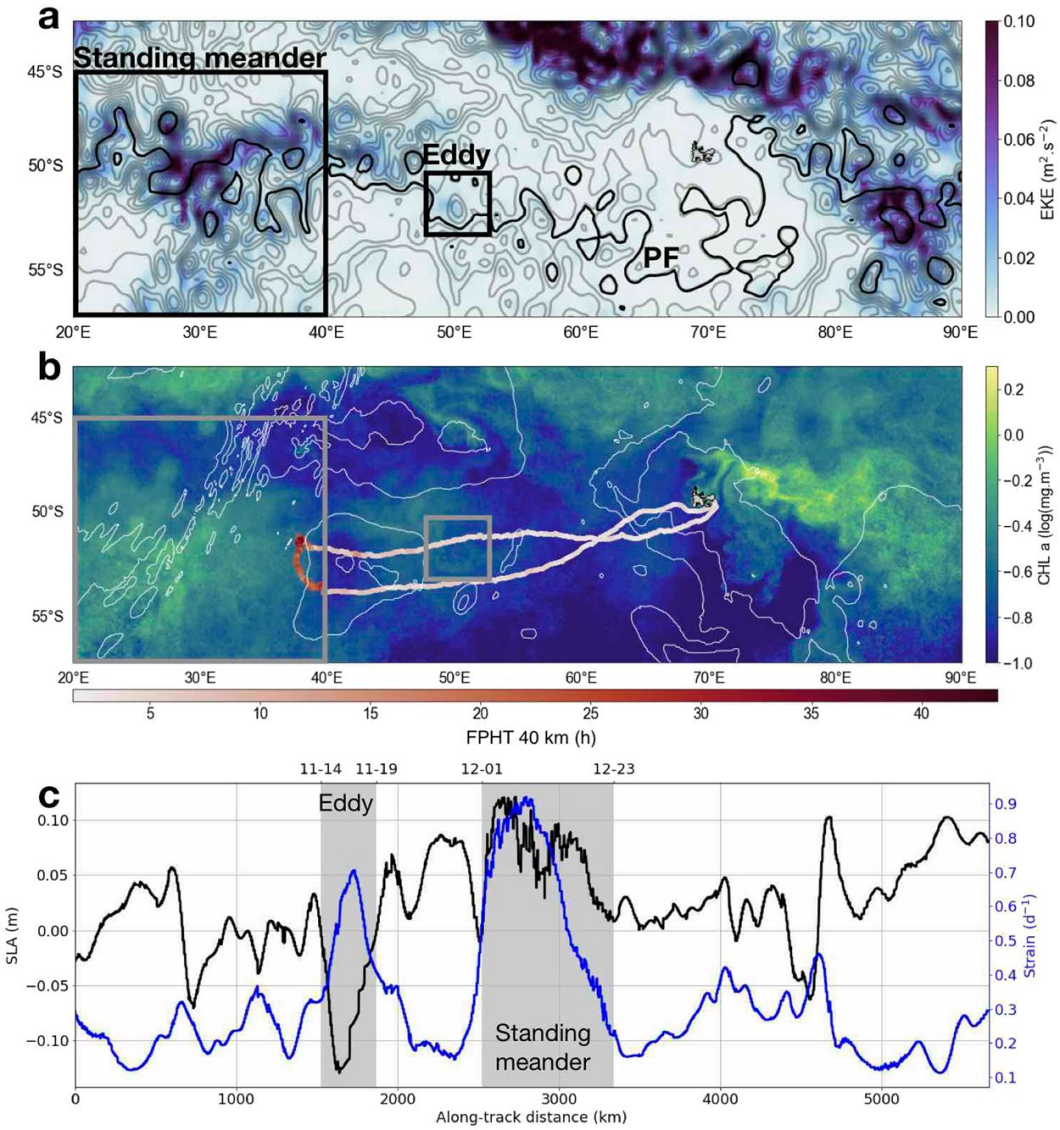
(**a**) Mean Eddy Kinetic Energy (EKE) over the seal’s journey (27 Oct 2014 - 21 Jan 2015) superimposed with sea surface height contours at mid-trajectory (12 Dec 2014) ranging from −1.6 to 1.7 m with 0.1 m increments obtained from AVISO satellite data. The Polar Front (PF) is indicated in bold black as the −0.61 m contour according to Kim and Orsi^70^. (**b**) Mean chlorophyll a concentration obtained from satellite data during the seal’s journey superimposed with the seal’s trajectory colored by the First-Passage Hunting Time (FPHT) with a radius R = 40 km, i.e. of the order of the first Rossby radius of deformation in the Kerguelen area. 2 and 4 km bathymetry contours from ETOPO5 are shown in white. On (**a** and **b**) black and white rectangles identify the standing meander area and cyclonic eddy discussed in the main text. (**c**) Lagrangian time series of Sea Level Anomaly (SLA, in black) and strain (in blue) along the seal’s path derived from satellite data (see Methods).

The Antarctic Circumpolar Current (ACC) hosts a small number of standing meanders localized in the lee of topographic features. These meandering large-scale jets trigger mesoscale eddies, with a size of 50 to 200 km, associated with hotspots of eddy kinetic energy of up to two orders of magnitude greater than in surrounding areas where the eddy activity is weak^1–4^. Thompson *et al*.^2^ reported four such standing meander regions across the ACC, among which one is localized between 45–60°S/20–40°E (Fig. 1). This standing meander area, linked to the topographical feature of the Southwest Indian Ridge, is identifiable from climatological altimetry data^2,5^ and has an average eddy kinetic energy value greater than 0.10 m^2^ s^−2^ over the duration of our study (from October 2014 to January 2015) (Fig. 2a).

Dynamical studies of the last decade further indicate that the flow shear, hereafter referred to as the strain field, associated to these mesoscale eddies generates submesoscale fronts with a size of 5 to 20 km in-between them and on their edges^6–8^. These structures, mainly thermohaline fronts, are now thought to capture most of the vertical velocities in the upper ocean and therefore to be the preferential pathway for the vertical exchange of heat, nutrients and other tracers between the surface and the deep ocean. In the iron-limited Southern Ocean, these dynamics have profound implications for phytoplankton production and biogeochemical systems^9–11^. However, these submesoscale dynamics are not well documented and poorly quantified in the ocean due to the lack of submesoscale-resolving *in situ* observations available over large domains. As a consequence, impacts on upper trophic levels remain largely unexplored. While numerous studies have identified mesoscale eddies as favorable feeding grounds for top predators such as elephant seals^12–15^, the relation between submesoscale turbulence and marine top predator’s at-sea foraging behaviour has only been inferred from altimetry-derived Lagrangian diagnostics^16,17^. To date, this has been the only available approach to overcome the lack of bio-physical observations capable of resolving oceanic submesoscale features.

The present study focuses on the area west of the Kerguelen Islands (Indian sector of the Southern Ocean, Fig. 2) over the austral summer. The Kerguelen region is known to host a complex local circulation^18,19^ due to the presence of strong bathymetric features, e.g. the Kerguelen Plateau and the Southwest Indian Ridge (Fig. 2b). These features are likely to play a positive role for for the marine fauna. Indeed, the Kerguelen Islands are home to large colonies of marine predators and, in particular, host the second largest population of Southern Elephant Seals (SES, *Mirounga leonina*). The southern elephant seal is a deep-diving, wide-ranging marine predator species^20^ that forages in either one of three main habitats: the Antarctic continental shelf, the Kerguelen Plateau or deep open water regions^21^. Antarctic shelf waters provide prime habitat for both sexes, but females from Kerguelen use them less because advancing sea ice may impede their annual return to breed^21^. Instead, most female SES from Kerguelen forage in the open water regions and along the sea ice edge year round^13,14,22,23^. Over the last two decades, the post-breeding trips (from ∼October to January) of 103 females have been recorded as part of the National Observing System MEMO (Mammifére Echantillonnneurs du Milieu Océanique). More than 80% of these seals target the area east of the plateau (Fig. 1) where high eddy kinetic energy (>0.20 m^2^ s^−2^, Fig. 2a) and chlorophyll concentrations (Fig. 2b) are found. Alternatively, less than 10% of seals head west of Kerguelen Islands (Fig. 1) in a region of weak eddy kinetic energy (<0.02 m^2^ s^−2^, Fig. 2a) and low chlorophyll concentrations (Fig. 2b). However, feeding-like behaviour of southern elephant seals in the standing meander region west of Kerguelen Islands has previously been idenfied^24^. This particular region also appears as a biological hotspot for predators from adjacent colonies on Prince Edwards Islands (46.9°S, 37.7°E), including elephant seals^25,26^ and macaroni penguins^27^.

The present work complements the research of other tagging programs that use animals as oceanographers to study animal behaviour in relation to characteristic water masses^28^ and large- to meso-scale oceanic features^29–32^. However, this study focuses on submesocale physics thanks to a unique dataset at unprecedented high-resolution collected *in situ* by a female southern elephant seal from the Kerguelen Islands. Unlike previous tags mounted on marine mammal, the one used in this study recorded for the first time every single dive realized by a seal during its journey (or over 6900 dives). This dataset advantageously contains both physical and behavioural data, which allow us to explore the submesoscale dynamics of the region west of the Kerguelen Islands as well as its relation with the foraging behaviour of the tagged seal. The seal’s trajectory displays a similar excursion to the standing meander site as the ones discussed above. This excursion is accompanied by a significant increase in foraging behaviour up to 5 times greater than during the rest of the trip (Fig. 2b). Here, we propose for the first time a physical explanation for such a biologically active area based on the study of submesoscale dynamics in the vast domain sampled by the seal and broadly defined by 20–70°E/45–55°S, potentially explaining the other southern elephant seals western excursions observed in Fig. 1.

## Results

### Southern elephant seal observations at unprecedented high-resolution

Results are inferred from a unique dataset of physical and biological observations collected by a female southern elephant seal (Fig. 2b). The seal was equipped with sensors measuring temperature, conductivity and pressure at a continuous high-frequency of 0.5 Hz and its travel was tracked through the Argos satellite system. Buoyancy, which is of opposite sign to the fluid density, and spiciness, which indicates thermohaline variations along constant density surfaces^33^ (see Methods), are estimated from temperature and salinity observations. Both fields have a final vertical resolution of 1 m and a horizontal resolution of 1 km (see Methods). This gives access to a unique dataset of vertical sections (x–z) of buoyancy and spiciness at very high-resolution over a long distance (>5000 km) and down to 600 m in the ocean interior. Simultaneously, the same device recorded animal behavioural information, based on the premise a predator will maximize resource acquisition by adapting its movement in response to prey distribution and density^34,35^. More precisely, the seal’s foraging behaviour is estimated through the computation of the First-Passage Hunting Time (FPHT), which combines the first passage time metric used in Bailleul *et al*.^36^ and the sinuosity method of Heerah *et al*.^37^. FPHT advantageouly takes into account the horizontal and vertical dive’ sinuosity in order to indicate the amount of time the seal spent hunting within an area of given radius. In this study, FPHT is computed at the radius R of 40 km, which is of the order of the local Rossby radius of deformation in terms of wavelength^38^, in order to capture the mesoscale features of the area. However, a sensitivity analysis demonstrates that the results are robust to the choice of R (see Methods and Supplementary Fig. S1).

During its three-month post-breeding round trip from the Kerguelen Islands in the austral summer (28 Oct 2014 to 21 Jan 2015), the seal traveled a distance of 5665 km. The seal’s voyage can be divided into three distinct parts (Fig. 2b): two relatively straight transit lines back and forth between the Kerguelen Islands and the standing meander site–accounting for 85% of the total distance (4850 km) but only 67% of the total time–and the remaining 15% of the trip (815 km), accounting for a third of the time, spent in the standing meander region. The seal’s foraging activity appears to be enhanced at the standing meander site, where FPHT reach values up to five times that of the rest of the trip (Fig. 2b). In order to understand this contrast, we study the underlying physics at meso- and sub-mesoscale.

### Submesoscale dynamics west of the Kerguelen Plateau

During its 85 day trip, the seal encounters two well-defined mesoscale features: a cyclonic eddy and a meander located at the easternmost tip of the standing meander area (Fig. 2). The cyclonic eddy has a size of ∼150 km and is located north of an anticyclonic one, creating a dipole structure, which generates a westward jet of ∼0.2 m/s in between both eddies. The meander has an elongated shape with a length of ∼350 km and a width of ∼50 km. The local flow is directed southward with a magnitude of ∼0.3 m/s. In both structures, the seal travels in the same direction as the current. More precisely, the seal crosses the southern part of the cyclonic eddy, amounting to a cumulative distance of 350 km, in 6 days and corresponding to a speed of ∼60 km/day. In contrast, the seal spends 23 days on the edge of the meander over a cumulative distance of 815 km, which corresponds to a speed of ∼35 km/day, i.e. the seal reduces overall speed by roughly 40% in the meander in comparison with the cyclonic eddy. However, both mesoscale features, identified from satellite data of sea level anomaly (see Methods), are associated to a strong strain field reaching 0.8 day^−1^ in the standing meander and 0.6 day^−1^ in the cyclonic eddy (Fig. 2c). Since theoretical studies indicate that a turbulent mesoscale eddy field can drive and constrain submesoscale turbulence^7,39^, both areas are potentially favorable for the development of submesoscale features.

#### Standing meander

**Figure 3:**
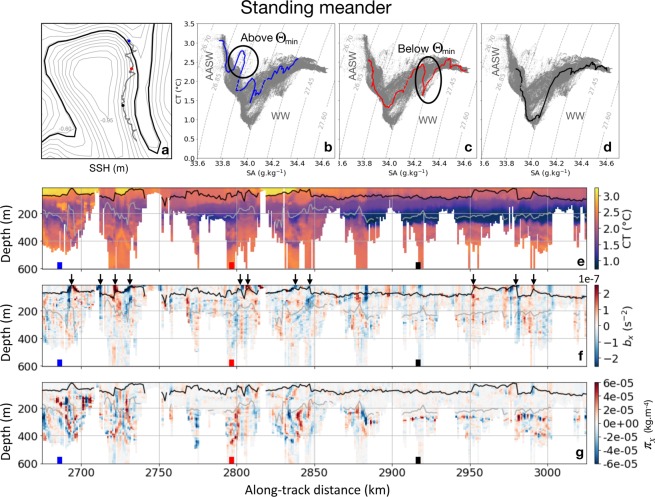
Key physical properties for a 350-km long section in the standing meander. (**a**) Snapshot of Sea Surface Height (SSH) on 2014/12/10 superimposed with the seal’s trajectory from 2014/12/5-14. Blue, red and black dots indicate the profiles in the Temperature (CT)–Salinity (SA) diagrams. The Polar Front is indicated in bold black as the −0.61 m contour according to Kim and Orsi^70^. Sea surface height contours are incremented by 0.05 m. (**b**) Temperature–salinity diagram highlighting a profile presenting intrusions above the temperature minimum (Θ_min_). (**c**) Temperature–salinity diagram highlighting a profile presenting intrusions below the temperature minimum. (**d**) Temperature–salinity diagram highlighting a profile presenting no intrusion. In (**b**–**d**), the gray profiles correspond to the rest of the transect in (**a**). (**e**) Vertical section of temperature for the transect in (**a**). (**f**) Vertical section of lateral gradient of buoyancy (b_x_) for the transect in (**a**). Black arrows indicate instances of mixed layer depth shoaling, concomitant with intense gradients of buoyancy. (**g**) Vertical section of lateral gradient of spiciness (π_x_) for the transect in a). In (**e**–**g**), the profiles shown in (**a**–**d**) are indicated by blue, red and black squares at the bottom of the plots. The black line indicates the mixed layer depth and the gray line the depth of the temperature minimum.

Seal observations clearly indicate that submesoscale buoyancy gradients with a width of 5 to 20 km are indeed present at the standing meander, extending from the surface down to 500 m and intensified within the mixed layer (Fig. 3f). A succession of large surface gradients coincides with instances of mixed layer depth shoaling on the edges of the meander as inferred from the comparison with sea surface height (black arrows in Fig. 3f). These events are presumably related to the seal zig-zagging through the standing meander and thus identify its edges. Gradient magnitudes reach values larger than 2 × 10^−7^ s^−2^, consistent with numerical and observational studies^40–43^. The mixed layer depth has an average value of 85 m (black line in Fig. 3e–g).

Similar patterns are observed for lateral gradients of spiciness, indicative of thermohaline intrusion occurring along isopycnals and thus of oceanic mixing. They also have a scale of 5 to 20 km and extend down to at least 500 m (Fig. 3g). However, the mixed layer is free of lateral gradients of spiciness, which are enhanced below the temperature minimum (see for example the gradient at the red square location in Fig. 3g). Altough, several instances of large lateral gradients of spiciness occur above the temperature minimum, especially noticeable from 2675 to 2750 km (Fig. 3g, blue square). Strong lateral gradients of spiciness reach values of 8 × 10^−5^ km^−4^. Finally, strong lateral gradients of spiciness are not always associated with buoyancy ones (see for example between 2780 and 2850 km, red square in Fig. 3f–g). This happens when anomalies of temperature and salinity are stretched by the local strain field, which generates strong gradients of temperature and salinity at submesoscale that are however density compensated.

**Figure 4:**
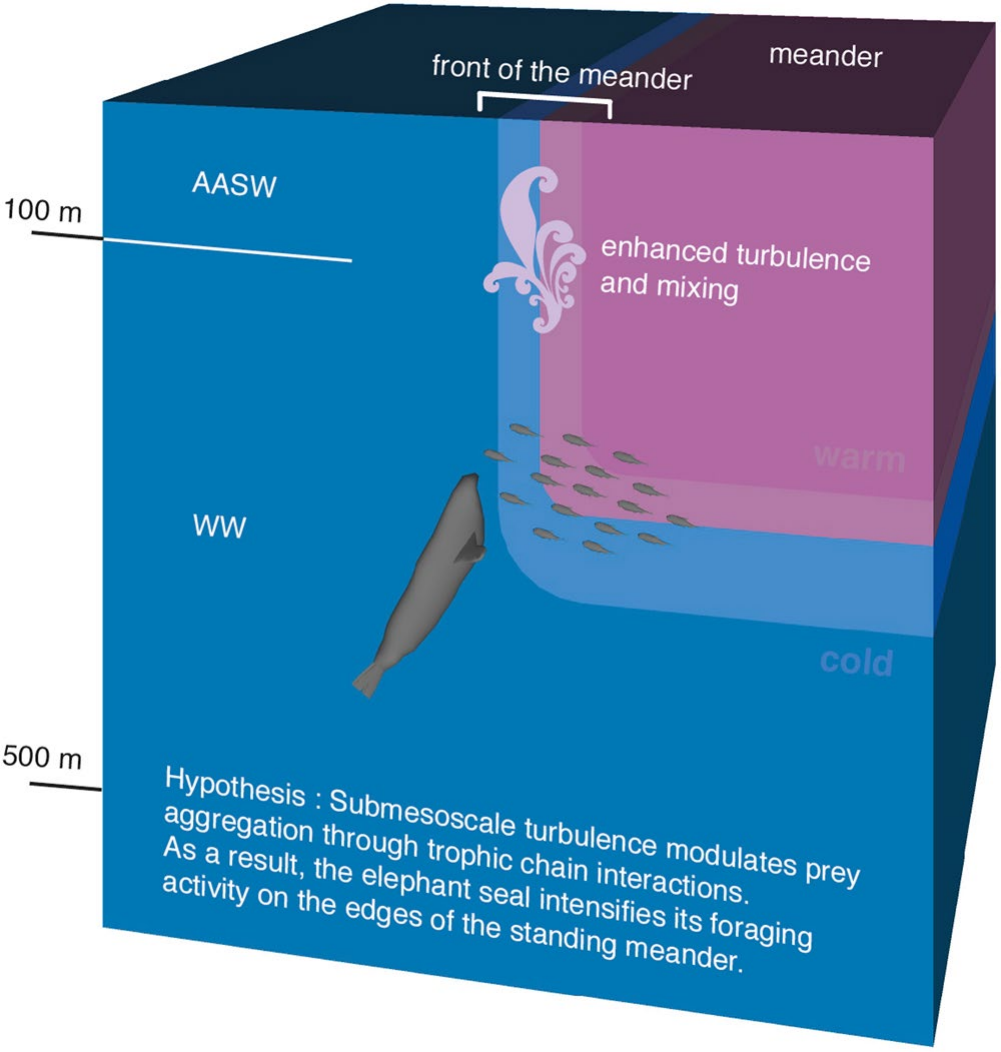
Schematic summarizing how submesoscale turbulence distributed on the edges of coherent mesoscale structures (here a standing meander of the ACC) affects the seal’s foraging behaviour and featuring the water masses encountered by the seal; Antarctic Surface Water (AASW) and Winter Water (WW). Relative depths of water masses are provided as an indication only. Tandi Reason Dahl is the author of this image.

To further understand these water mass intrusions, we use temperature–salinity diagrams (Fig. 3b–d). The seal mainly encounters Winter Water, formed during the previous cold season, and characterized by the presence of a subsurface temperature minimum and shallow, warm and fresh Antarctic Surface Water sitting mainly above Winter Water (highlighted in Fig. 3b–d and schematized in Fig. 4). Temperatures are comprised between 0.6 and 3.2°C. Temperature-salinity profiles present numerous thermohaline intrusions localized at different depths (Fig. 3b–e). As previously mentioned, intrusions occur above the temperature minimum, as highlighted on the blue profile in Fig. 3b and below the temperature minimum, as shown on the red profile in Fig. 3c. For comparison, Fig. 3d highlights a profile presenting no such intrusion. These intrusions, as well the erosion of the temperature minimum at depth, are also reflected in the vertical section of temperature (Fig. 3e) presenting sharp variations at submesoscale. Overall these results highlight the intense submesoscale activity present along the edges of the meander where waters are being mixed.

#### Cyclonic eddy

**Figure 5:**
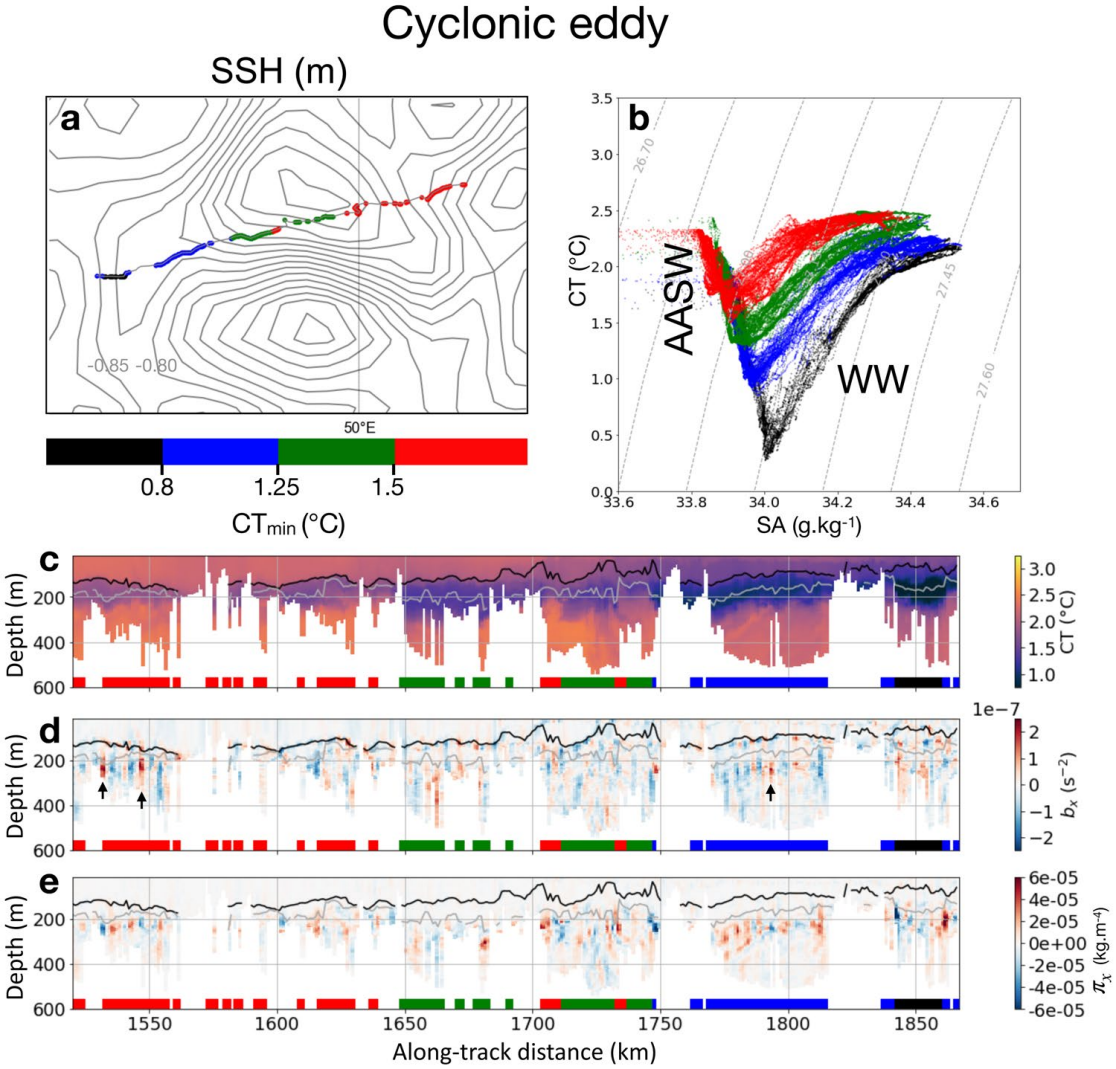
Key physical properties for the 350-km long section on the southern part of the cyclonic eddy. (**a**) Snapshot of Sea Surface Height (SSH) on 2014/11/16 superimposed with the seal’s trajectory from 2014/11/14-19 colored by the dives subsuface temperature minimum (CT_min_). Sea surface height contours are incremented by 0.05 m. (**b**) Temperature (CT)–Salinity (SA) diagram for the transect in a. Same color code as (**a**). (**c**) Vertical section of temperature for the transect in (**a**). (**d**) Vertical section of lateral gradient of buoyancy (b_x_) for the transect in (**a**). The black arrows highlight a few lateral gradients of buoyancy localized below the temperature minimum. (**e**)Vertical section of lateral gradient of spiciness (π_x_) for the transect in a). In (**c**–**e**), the profiles shown in (**a**,**b**) are indicated by the colored squares at the bottom of the plots. The black line indicates the mixed layer depth and the gray line the depth of the temperature minimum.

The seal-sampled water masses (Antarctic Surface Water and Winter Water, Fig. 5) are characterized by temperatures between 0.4 and 2.4 °C with warmer and colder waters north and south of the transect, respectively (Fig. 5a,b). The dives can be separated by temperature minima of 0.8, 1.25 and 1.5 °C. Lateral gradients of buoyancy below the mixed layer are of the same order as those observed in the meander (Fig. 5d). However, a few differences are worth mentioning. First, the mixed layer is deeper in the cyclonic eddy with an average value of 115 m and, unlike in the meander, no lateral gradients of buoyancy are observed within it. More importantly, the cyclonic eddy is characterized by weak lateral gradients of spiciness seldom reaching 2 × 10^−5^ kg m^−4^ (Fig. 5e) and systematically localized below the temperature minimum (see for example the black arrows in Fig. 5d). The absence of thermohaline intrusion is also reflected in the temperature–salinity diagram (Fig. 5b) that displays a remarkable fan-shape distribution with gaps between the different temperature minima, indicative of the clear separation between water types, and thus, stratification and absence of mixing.

#### Entire transect

**Figure 6:**
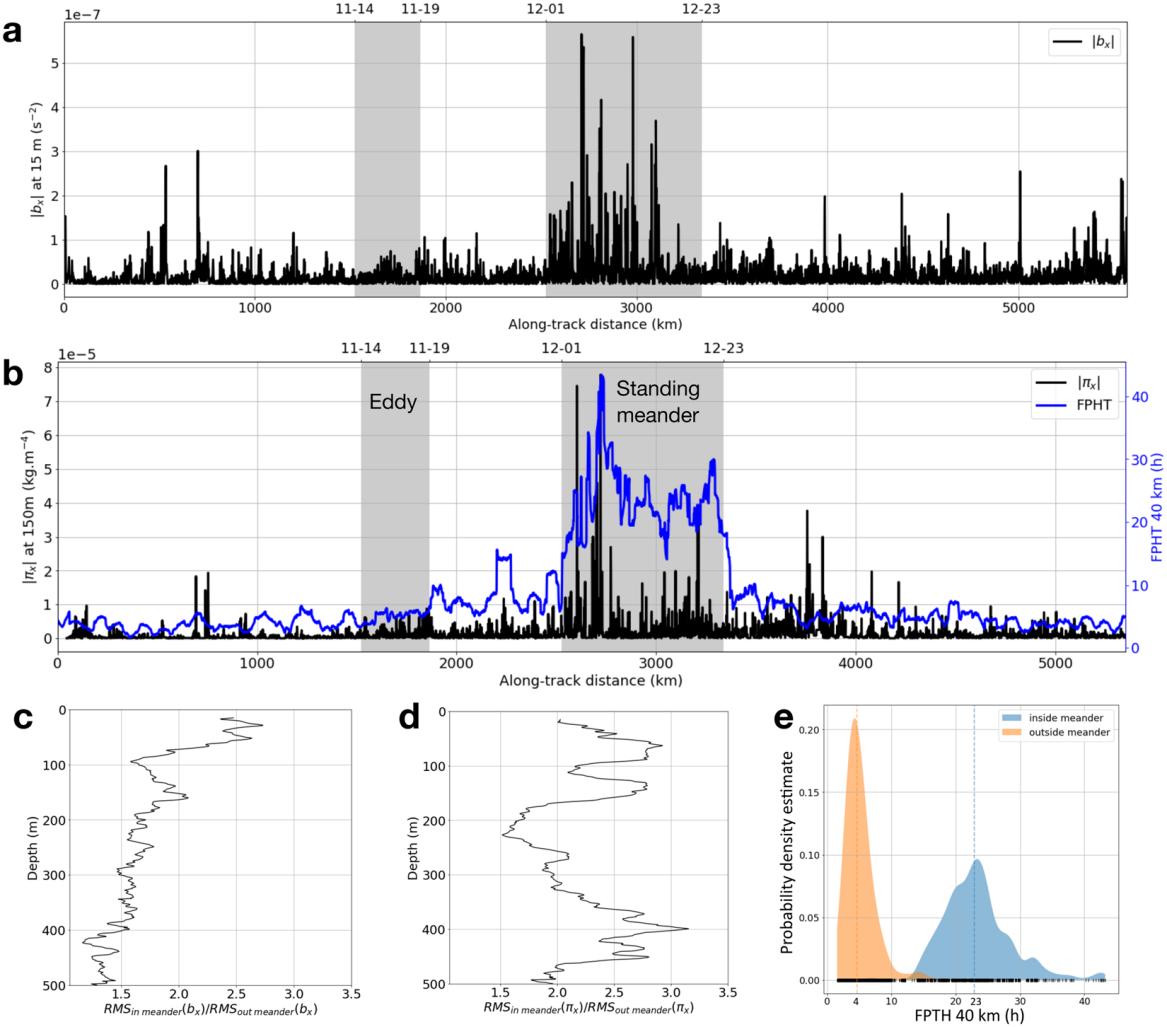
Lagrangian time series along the seal’s track of (**a**) Lateral buoyancy gradient (|b_x_|) at 15 m. (**b**) Lateral gradient of spiciness (|π_x_|) at 150 m (in black) and First-Passage Hunting Time (FPHT) at 40 km (in blue) calculated as in Fig. 2b. The eddy and standing meander discussed in the main text and described in Figs 5 and 3 are indicated in gray. Root mean square (RMS) ratio inside/outside of the meander as a function of depth for (**c**) lateral buoyancy gradient. (**d**) lateral gradient of spiciness. (**e**) Probability density estimate of First-Passage Hunting Time (FPHT, in hour) inside (blue) and outside (orange) of the meander. Medians are indicated in dashed colored lines and a rug plot is shown on the bottom. Lateral gradients of buoyancy and spiciness, and First-Passage Hunting Time are significantly greater inside the meander.

Overall, the submesoscale characteristics along the entire trajectory (standing meander excluded) resemble those found in the cyclonic eddy; inferred from the time series of lateral gradients of buoyancy and spiciness presented in Fig. 6a,b. Lateral gradients of buoyancy within the mixed layer (shown at 15 m in Fig. 6a), and spiciness throughout the water column (shown at 150 m in Fig. 6b), are stronger inside than outside the standing meander. However, below the mixed layer, lateral gradients of buoyancy are homogeneous along the entire transect, with no clear intensification in the meander (not shown). In the first 80 m, the root mean square of the lateral gradient of buoyancy inside the standing meander is greater than outside of it by a factor >2. Below 80 m, this factor is <2 and further decreases with depth (Fig. 6c). For lateral gradients of spiciness, the root mean square inside the standing meander is 1.5 to 3.2 times greater than outside the standing meander (Fig. 6d), highlighting the strong mixing occurring in the vicinity of the standing meander. Overall, the observed submesoscale features are mostly located in regions of intense shear, consistent with theoretical studies^7,39^ indicating that submesoscale frontal structures result from the mesoscale stirring field generated by co-interacting eddies.

### Impact of submesoscale dynamics on southern elephant seal behaviour

The intense submesoscale fronts observed at the standing meander site suggest that the biological pump is stimulated via mixing and frontogenesis processes that contribute to the injection of nutrients from deeper layers to the euphotic zone where primary production occurs^11^. However, knowing how marine top predators respond to this submesoscale turbulence remains an open question.

Here, we investigate the seal’s foraging activity and, in particular, relate submesoscale fronts to local foraging behaviour. To do so, we identify transitions in movement patterns (e.g. from transit behaviour to feeding) between mesoscale regions of 50 to 200 km-size along the seal’s trajectory. As previously mentioned, we use the First-Hunting Passage Time (FPHT) with a radius R of 40 km, which captures the mesoscale features of the area, as a proxy for the seal’s foraging activity (see Methods).

FPHT intensifies significantly at the standing meander site with values >20 hours. The bimodal distribution of FPHT inside and outside of the standing meander further underscores this finding, with FPHT between 15 and 40+ hours inside the standing meander, while generally being <10 hours outside of it with a median of 4 hours outside versus 23 hours inside (Fig. 6e). There is a good agreement between the time series of FPHT and both lateral gradient of spiciness at 150 m and lateral gradient of buoyancy at 15 m (Fig. 6a,b). This is particularly noticeable at the standing meander where strong gradients of buoyancy and spiciness are associated with greater FPHT (Fig. 6). For lateral gradients of spiciness, different depths lead to similar results. On the other hand, for lateral gradients of buoyancy, this results holds in the first 80 meters but not below. This is because at depth, lateral gradients of buoyancy are more homogeneously distributed along the seal’s trajectory (Fig. 6c).

**Figure 7:**
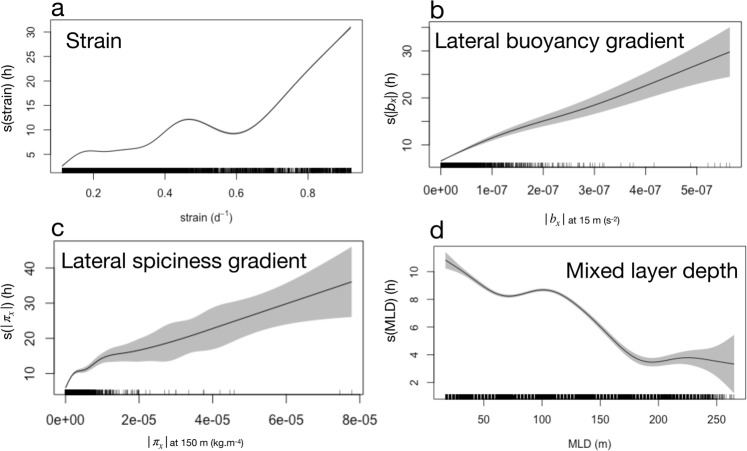
Effect of key physical properties on the seal’s foraging effort, quantified by First-Passage Hunting Time (FPHT) at 40 km, inferred from generalized additive models. (**a**) Strain derived from satellite data. (**b**) Lateral buoyancy gradient (|b_x_|) at 15 m derived from seal data. (**c**) Lateral spiciness gradient (|π_x_|) at 150 m derived from seal data. (**d**) Mixed Layer Depth (MLD) derived from seal data. Shaded grey polygons show 95% confidence interval. A rug plot is added to the bottom of each panel.

Generalized additive models corroborate the qualitative relationships observed between FPHT and key physical quantities (Figs 2 and 6). At mesoscale, FPHT is positively linked to the strain field (Fig. 7a), consistent with previous studies suggesting that elephant seals target energetic mesoscale structures^16,17,21^. At submesoscale, FPHT is also positively linked to lateral gradients of buoyancy at 15 m and lateral gradients of spiciness at 150 m (Fig. 7b,c). This last result highlights the seal’s preference for submesoscale features, that are concentrated at the standing meander site. Moreover, the mixed layer depth is negatively linked to FPHT, highlighting the seal’s preference for shallower mixed layer, which often occur on the edges of mesoscale structures, and thus correspond to areas of strong buoyancy gradients (Figs 3f and 7c). However, it is hard to disentangle the seasonal component from the spatial variability at submesoscale in the time series of the mixed layer depth.

These results emphasize that submesoscale frontal structures constitute favorable foraging habitats for top marine predators such as elephant seals (Fig. 4). The seal’s foraging activity is significantly enhanced on the edges of the standing meander where most of the submesoscale features are located. The standing meander west of the Kerguelen Islands that hosts the vast majority of submesoscale features thus appears to be a physical and biological hotspot for apex marine predators.

## Conclusion and Discussion

Our results provide evidence that submesoscales, while dominant in winter time, are also active in the Southern Ocean during the summertime. This is consistent with recent observational findings obtained from gliders in the Drake passage in summer^43^ and in the sub-Antarctic zone in spring^42^.

This work documents the spatial distribution of submesoscale features over a large oceanic domain (>5000 km) in the under sampled area west of the Kerguelen Islands. A clear partitioning of the physical properties at submesoscale is observed: within the standing meander area, the mixed layer contains strong lateral gradients of buoyancy. Below the mixed layer, intense lateral gradients of spiciness are present down to 500 m, indicating the presence of strong thermohaline intrusions, also noticeable on temperature–salinity diagrams (Fig. 3). In comparison, the rest of the area, including the cyclonic eddy discussed in the results section, is characterized by weak lateral gradients of spiciness throughout the water column and tame lateral gradients of buoyancy within the mixed layer, which is deeper in the cyclonic eddy than in the meander. However, lateral gradients of buoyancy at depth are comparable and relatively weak over the entire domain. The weakness of these lateral gradients of buoyancy can be understood through their link with the mesoscale strain field. Indeed, the strain field associated to mesoscale eddies is known to stretch buoyancy (or density) anomalies, leading to the creation of strong gradients at submesoscale^7^. However, over the vast domain sampled by the seal, the mesoscale strain field is weak (apart in the standing meander and the cyclonic eddy), as inferred from the map of eddy kinetic energy (Fig. 2a) and the time series of the strain field (blue curve in Fig. 2c). As a consequence, lateral gradients of spiciness and buoyancy remain subdued in most of the domain. However, this contrast is also observed between the two distinct mesoscale features encountered by the seal: the standing meander and the cyclonic eddy. One simple hypothesis explaining this dichotomous distribution may be related to their respective sizes. While the meander is an elongated feature of ∼350 km of length, the cyclonic eddy has a smaller size of ∼100 km, which makes it statistically less likely to host mixing events and thus even less likely to observe it. Furthermore, our observational results confirm modelling studies of the last decade showing that submesoscale fronts are produced mostly at the edges and in-between mesoscale eddies^39^.

The abundance and strength of submesoscale features observed on the edges of the standing meander are of biological significance for upper trophic levels: the SES considered in this study spends significantly more time foraging in the vicinity of strong submesoscale features located on the edge of the meander where it also decreases its speed. This is consistent with the results of Della Penna *et al*.^17^, who found that SES adopt a “quasi planktonic behaviour”, i.e. are horizontally advected, on the edge of mesoscale features identified as favorable feeding grounds. At these locations, an intense foraging activity is however observed on the vertical, similar to the animal considered in this study. Our results are also consistent with recent findings of Hindell *et al*.^21^ suggesting that rather than targeting one specific water mass, SES may simply be targeting areas of high mixing that are presumably concomitant with high prey concentrations due to the higher turnover of nutrients within the water column. Our findings are also consistent with previous work linking mesoscale features to SES behaviour. Indeed, numerous studies of the last decade have identified mesoscale eddies as favorable feeding grounds for SES with a preference for cold cyclonic structures and the edges of anticyclonic ones^12–15^. Both structures were reported to be enriched in organisms of different trophic levels^44–46^, and aggregate resources into narrower layers closer to the surface where they are more accessible to air-breathing SES^47^. Interestingly, when the seal encounters the standing meander, it most intensified his foraging behaviour at a depth of 220 m (Supplementary Fig. S2). Furthermore, hunting dive depth and maximum dive depth variances decrease (Supplementary Fig. S2). This relatively constant foraging dive depth of SES possibly reflects narrower prey fields associated with improved foraging success^24,48^, hinting that local dynamics creates predictable biological boundaries that facilitate prey accessibility.

Since the beginning of the bio-logging program in 2004, all but one of the five post-breeding females trajectories located west of the Kerguelen Islands present similar excursions to the standing meander site (Fig. 1). Even though the reason why SES transit west is presumably related to multiple factors, such as minimizing intra-specific competition^49^ for instance, it is still necessary that they find optimal conditions for feeding. This suggests that western excursions to the standing meander region (Fig. 1) are not accidental, and may be explained by the recurrently elevated physical activity of the site. Furthermore, individual SES often return to the same broad scale foraging grounds over consecutive years, presumably because these habitats are reliable ‘oasis’ in a highly variable environment^50,51^. This site loyalty has been shown to be an effective strategy adopted by marine predators foraging in a dynamic and heterogeneous environment such as the Southern Ocean^50,52,53^. These findings are consistent with the fact that the standing meander region highlighted in this study is a permanent physical feature of the Southern Ocean, as described by Thompson *et al*.^2^. As such, the western trips observed in Fig. 1 are unlikely a temporary phenomenon, and are expected to be repeated by individuals as elephant seals have a high degree of individual foraging site fidelity over periods of up to 10 years^50,54^.

Within the Antarctic Circumpolar Current, other standing meanders and areas of high eddy kinetic energy may also act as physical and biological hotspot. For instance, the region east of the Kerguelen Islands located at ∼80°E–50°S, analyzed in Siegelman *et al*. (submitted), is a homogeneous hotbed of eddy kinetic energy where a strongly turbulent mesoscale eddy field generates intense submesoscale motions associated to a vigorous vertical velocitiy field. In addition to the iron input from the Kerguelen plateau which sustains a recurrent plume of primary production near shore^9^, Siegelman *et al*. (submitted) show that the submesoscale turbulence, and its associated vigorous vertical velocities, may stimulate primary production in the open ocean. The combined intensity of submesoscale features, boosting the biological system and modulating prey aggregation^15^, and its proximity to the Kerguelen Islands may thus explain southern elephant seals’ statistical preference for the high eddy kinetic energy area east of the Kerguelen plateau (Figs 1 and 2) or maybe because the western standing meander region is much farther away.

Our study, limited to a region of the Southern Ocean, stresses the need for more submesoscale-resolving physical and biological observations across the globe and during different seasons. In particular, it would be interesting to repeat this analysis during wintertime when submesoscale features are known to be more abundant and energetic^55^. Ultimately, understanding how the behaviour of individual marine predators is modulated by surrounding structures is key for assessing the health and functioning of open ocean ecosystems and is instrumental in designing effective marine protection policies in a changing climate.

## Methods

### Satellite data

85 daily maps of gridded 0.25° × 0.25°L4 Sea Surface Height (SSH) and Seal Level Anomaly (SLA) were obtained from the AVISO Ssalto/Duacs products, covering the spatial and temporal extent of the seal observation data. The Lagrangian time series of SLA along the seal’s track was used to identify the standing meander site (the region of positive SLA between 2525 and 3340 km) and the cyclonic eddy (the region of negative SLA between 1520 and 1865 km) (Fig. 2).

Using the geostrophic approximation, geostrophic surface currents (u, v) are derived as

$$u=\frac{g}{f}\frac{\partial SSH}{\partial x},v=-\frac{g}{f}\frac{\partial SSH}{\partial y},$$

where g is gravity and f the Coriolis parameter^56^. The strain field σ is subsequently computed as

$$\sigma =\sqrt{{({u}_{x}-{v}_{y})}^{2}+{({v}_{x}+{u}_{y})}^{2}},$$

where subscripts denote partial derivative.

In addition, the map of Chlorophyll a concentration level 2 data in Fig. 2b has been processed by CLS for the Kerguelen area. It corresponds to a time average of Chlorophyll a concentration over the 85 days of the seal’s journey.

### Southern Elephant Seal dataset

High-resolution data was recorded by a seal-borne Conductivity-Temperature-Depth Satellite Relay Data Logger (CTD SRDL, Sea Mammal Research Unit, University of St Andrews) deployed on a post-breeding female southern elephant seal from the Kerguelen Islands (49°20′S, 70°20′E). The seal is tracked by the Argos satellite system and is equipped with sensors recording conductivity, temperature and pressure at a continuous frequency of 0.5 Hz between 27 October 2014 and 20 January 2015, with an accuracy of ±0.02 °C for temperature and ±0.03 g/kg for salinity^57^. Only the ascending phase of a dive is used because it is more uniform in speed and direction compared to the descent when the seal dives sinuously to forage. The dataset is comprised of 6942 dives, or over eighty dives per day, which corresponds to a cumulative length of 5665 km with a median spacing between two dives of 700 m (Supplementary Fig. S3). Dives can be as deep as 500 to 1000 m. They generally last less than 25 minutes and are separated by a few minutes surfacing, where the seal breathes without transiting. More than 85% of the dives reach a depth of at least 100 m, 45% reach 300 m and 25% are 400 m or deeper.

To ensure a better accuracy of the conductivity-derived salinity data, two additional steps are applied to the temperature and salinity fields. First temperature and salinity are corrected for a thermal cell effect, and then a density inversion removal algorithm is applied to the salinity field. Potential density is then calculated from corrected temperature and salinity with the TEOS-10 equation^58^. The correction procedure and accuracy of the dataset are presented in more details in Siegelman *et al*.^57^. The dataset has been made available to the community and can be found on the Marine Mammals Exploring the Oceans Pole to Pole database (http://www.meop.net/).

The animal in this study was handled in accordance with the Institut polaire francais Paul-Emile Victor (IPEV) ethical and Polar Environment Committees guidelines as part of the SNO-MEMO and IPEV program 109 (PI. H. Weimerskirch). The experimental protocols were approved by the Ethics Committee of IPEV and Polar Environment Committees.

### Buoyancy

Along-track time series of buoyancy (in s^−2^), b = g(1 − ρ/ρ_0_), where g is gravity, ρ is potential density, and ρ_0_ = 1025 kg m^−3^ is a reference density reveals variability covering both meso- and submesoscales. For the analysis, in particular the calculation of lateral buoyancy gradients (b_x_), buoyancy was first linearly interpolated along the seal’s path onto a regular grid of 100 m resolution, corresponding to the shortest along-track distance between two dives (Fig. S1). A moving average with a 1 km window was then applied such that the final dataset has a vertical resolution of 1 m and a horizontal resolution of 1 km. Buoyancy anomalies are resolved by multiple vertical profiles, such that the structures are not related to aliasing of the along-track data.

### Spiciness

Spiciness (in kg m^−3^) is a state variable most sensitive to isopycnal thermohaline variations and least correlated with the density field^33^. Spiciness is conserved in isentropic motions and its value increases with increasing temperature and salinity. Positive (warm, salty) and negative (cold, fresh) subsurface spiciness anomalies can be generated by subsurface isopycnal advection across spiciness fronts^59,60^. As such, variation in spiciness is particularly useful to detect thermohaline intrusions characteristic of intense mixing. Spiciness is derived from salinity and temperature with the TEOS-10 equation^58^.

### Mixed layer depth

The mixed layer depth (in m) is defined as the level of a 0.03 kg.m^−3^ density increase with respect to the density at 15 m depth^61^.

### Quantify seal foraging activity

Foraging activity is estimated from high-resolution dive data, recorded at the continuous sampling frequency of 0.5 Hz, by taking into account both the horizontal and vertical sinuosity of a dive. Indeed, a seal is expected to decrease its speed when feeding and move more sinuously along the horizontal axis, displaying what is effectively area-restricted search behaviour (ARS)^13,62^. However, adding the vertical dimension is also important^36^, and high-resolution dive data of female SES demonstrates how vertical sinuosity can significantly improve the predictive capacity of ARS as a proxy for foraging success^24^. This is backed by simulated diving tracks of beluga whales (*Delphinapterus leucas*) used to refined ARS in a 3D space^63^.

**Figure 8:**
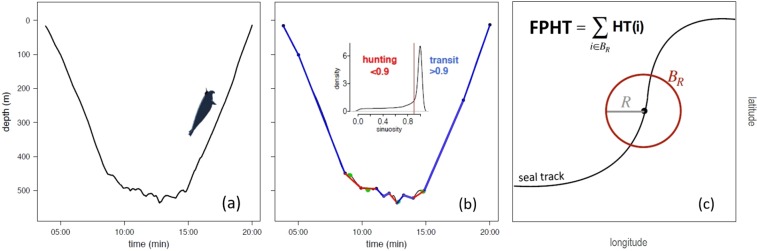
Schematic of First Passage Hunting Time (FPHT) calculation. (**a**) Typical dive profile recorded by the device deployed on the elephant seal. (**b**) The dive is separated into segments of hunting (red) and transit (blue) using the broken stick method (for details see Heerah *et al*.^37^). Hunting time (HT) is the summed total time of hunting segments in each dive. The number of prey encounter events detected by an on-board accelerometer (green) increased almost fourfold in hunting segments. (**c**) The First Passage Hunting Time at each point of the seal’s trajectory is then calculated as the sum of Hunting Time within a radius R.

Here, we develop an index, the First-Passage Hunting Time (FPHT), which indicates the amount of time spent hunting in a region of given radius and includes the following steps. First, we compute the Hunting Time (HT) via an automated broken stick algorithm, which summarizes the vertical sinuosity of the dive data based on the optimized number of segments within each dive. Further details outlining this step are provided in Heerah *et al*.^37^. A behavioural state is then assigned to each dive segment based on visual inspection of dive segment sinuosity distribution: low sinuosity values (>0.9) represent transit behaviour and high sinuosity values (<0.9) represent hunting behaviour. To validate this method, prey encounter events were detected from an accelerometer^64^ that, unfortunately, only recorded during the first three weeks of the seal’s trip due to limited storage capacity. However, evidence suggests that elephant seals even exhibit high foraging success during the outward transit when movement was relatively rapid and direct^65^. Furthermore, most prey encounter events (79%) recorded during these initial three weeks occurred within hunting segments, which comforts the use of the accelerometer data as validation data thoughouth the entire seal’s journey. This result is also consistent with the analyses of two post-breeding elephant seal trips in Heerah *et al*.^37^. Next, we modify the First-Passage Time approach (FPT)^66^, which is a scale-dependent foraging metric (i.e. ARS) that estimates how much time is required for an animal to cross a given radius. Instead, we estimate how much time spent hunting is required for the seal to cross a given radius, referred hereafter as First-Passage Hunting Time (FPHT). Values of FPHT and FPT are similar across scales, but differences demonstrate how FPT cannot always account for vertical foraging activity (Fig. S1). Relatively speaking, FPHT > FPT is where more time is spent hunting within dives than is captured by the seal’s horizontal movement and vice versa. This methodology is refined compared to Bailleul *et al*. (2008) who took the entire bottom phase time. Specifically, we sum the time spent in hunting segments and ignore the time spent in transit segments. A summary of FPHT derivation is presented in Fig. 8.

FPHT is computed at the radius R of 40 km in order to capture the mesoscale features of the area. However, results are robust to the choice of R. Indeed, although the behavioural pattern seems ambiguous at fine scale (r = 5 km), it is generally consistent at broader scales (r > 10 km): the seal transits west of Kerguelen for a month (low FPHT), followed by increased hunting activity at the standing meander (high FPHT), followed by a three-week transit return to Kerguelen (low FPHT) (Fig. S1).

### Generalized additive models

Generalized Additive Models (GAMs) were used to explore relationships between key physical variables and FPHT using a Gaussian distribution with the identity link function. Times series of FPHT at 40 km were used as the response variable, and time series of the lateral gradient of buoyancy at 15 m, lateral gradient of spiciness at 150 m and mixed layer depth (from the seal’s observations) and strain field (from satellite data) were used as explanatory variables. A summary table is presented in Supplementary Table S1. Model assumptions pertaining to GAMs, including normality and homogeneity of variance were checked using plots of residuals against fitted values^67,68^. GAMs were computed with the ‘mgcv’ package for R^69^.

## Supplementary information

Supplementary Information

## Data Availability

The marine mammal data were collected and made freely available by the International MEOP Consortium and the national programs that contribute to it (http://www.meop.net). The Ssalto/Duacs altimeter products were produced and distributed by the Copernicus Marine and Environment Monitoring Service (CMEMS) (http://www.marine.copernicus.eu).

## References

[1] Zika JD (2013). Vertical eddy fluxes in the southern ocean. J. Phys. Oceanogr..

[2] Thompson AF, Naveira Garabato AC (2014). Equilibration of the antarctic circumpolar current by standing meanders. J. Phys. Oceanogr..

[3] Rosso I, Hogg AM, Kiss AE, Gayen B (2015). Topographic influence on submesoscale dynamics in the southern ocean. Geophys. Res. Lett..

[4] Youngs MK, Thompson AF, Lazar A, Richards KJ (2017). Acc meanders, energy transfer, and mixed barotropic–baroclinic instability. J. Phys. Oceanogr..

[5] Kostianoy AG, Ginzburg AI, Lebedev SA, Frankignoulle M, Delille B (2003). Fronts and mesoscale variability in the southern indian ocean as inferred from the topex/poseidon and ers-2 altimetry data. Oceanology C/C of Okeanologiia.

[6] Klein P (2008). Upper ocean turbulence from high-resolution 3d simulations. J. Phys. Oceanogr..

[7] Klein P, Lapeyre G (2009). The oceanic vertical pump induced by mesoscale and submesoscale turbulence. Annu. review. marine science.

[8] McWilliams, J., Colas, F. &Molemaker, M. Cold filamentary intensification and oceanic surface convergence lines. *Geophys. Res. Lett*. **36** (2009).

[9] Blain S (2007). Effect of natural iron fertilization on carbon sequestration in the southern ocean. Nature.

[10] Perruche C, Rivière P, Lapeyre G, Carton X, Pondaven P (2011). Effects of surface quasi-geostrophic turbulence on phytoplankton competition and coexistence. J. marine research.

[11] Lévy, M., Ferrari, R., Franks, P. J., Martin, A. P. &Rivière, P. Bringing physics to life at the submesoscale. *Geophys. Res. Lett*. **39** (2012).

[12] Campagna C, Piola AR, Marin MR, Lewis M, Fernández T (2006). Southern elephant seal trajectories, fronts and eddies in the brazil/malvinas confluence. Deep. Sea Res. Part I: Oceanogr. Res. Pap..

[13] Bailleul F, Cotté C, Guinet C (2010). Mesoscale eddies as foraging area of a deep-diving predator, the southern elephant seal. Mar. Ecol. Prog. Ser..

[14] Dragon A-C, Monestiez P, Bar-Hen A, Guinet C (2010). Linking foraging behaviour to physical oceanographic structures: Southern elephant seals and mesoscale eddies east of kerguelen islands. Prog. Oceanogr.

[15] Abrahms B (2018). Mesoscale activity facilitates energy gain in a top predator. Proc. R. Soc. B.

[16] Cotté C, d’Ovidio F, Dragon A-C, Guinet C, Lévy M (2015). Flexible preference of southern elephant seals for distinct mesoscale features within the antarctic circumpolar current. Prog. Oceanogr.

[17] Della Penna, A., De Monte, S., Kestenare, E., Guinet, C. & d’Ovidio, F. Quasi-planktonic behavior of foraging top marine predators. *Sci. reports* **5** (2015). 10.1038/srep18063 PMC4678296 26666350

[18] Park Y-H, Fuda J-L, Durand I, Garabato ACN (2008). Internal tides and vertical mixing over the kerguelen plateau. Deep Sea Research Part II: Topical Studies in Oceanography.

[19] Bestley, S. *et al*. Ocean circulation and frontal structure near the southern kerguelen plateau: the physical context for the kerguelen axis ecosystem study. *Deep Sea Research Part II: Topical Studies in Oceanography* (2018).

[20] Hindell M, Slip D, Burton H (1991). The diving behavior of adult male and female southern elephant seals, mirounga-leonina (pinnipedia, phocidae). Aust. J. Zool..

[21] Hindell MA (2016). Circumpolar habitat use in the southern elephant seal: Implications for foraging success and population trajectories. Ecosphere.

[22] Bailleul F (2007). Successful foraging zones of southern elephant seals from the Kerguelen Islands in relation to oceanographic conditions. Philos. Transactions Royal Soc. B: Biol. Sci..

[23] Labrousse S (2017). Under the sea ice: Exploring the relationship between sea ice and the foraging behaviour of southern elephant seals in east antarctica. Prog. Oceanogr.

[24] Vacquié-Garcia J (2015). Predicting prey capture rates of southern elephant seals from track and dive parameters. Mar. Ecol. Prog. Ser..

[25] Massie PP (2016). The role of eddies in the diving behaviour of female southern elephant seals. Polar Biol..

[26] Tosh, C. A. *et al*. Marine habitats of juvenile southern elephant seals from marion island. In *SCAR XXXII & Open Science Conference* (2012).

[27] Whitehead TO, Kato A, Ropert-Coudert Y, Ryan PG (2016). Habitat use and diving behaviour of macaroni Eudyptes chrysolophus and eastern rockhopper E. chrysocome filholi penguins during the critical pre-moult period. Marine Biol..

[28] Biuw M (2007). Variations in behavior and condition of a southern ocean top predator in relation to *in situ* oceanographic conditions. Proc. Natl. Acad. Sci..

[29] Charrassin J-B, Park Y-H, Maho YL, Bost C-A (2002). Penguins as oceanographers unravel hidden mechanisms of marine productivity. Ecol. Lett..

[30] Boehme L, Thorpe S, Biuw M, Fedak M, Meredith M (2008). Monitoring drake passage with elephant seals: Frontal structures and snapshots of transport. Limnol. Oceanogr..

[31] Charrassin J-B (2008). Southern ocean frontal structure and sea-ice formation rates revealed by elephant seals. Proc. Natl Acad. Sci..

[32] Gove JM (2016). Near-island biological hotspots in barren ocean basins. Nat. communications.

[33] Flament P (2002). A state variable for characterizing water masses and their diffusive stability: spiciness. Prog. Oceanogr.

[34] Charnov EL (1976). Optimal foraging, the marginal value theorem. Theor Popul Biol.

[35] Fauchald P, Erikstad KE, Skarsfjord H (2000). Scale-dependent predator–prey interactions: the hierarchical spatial distribution of seabirds and prey. Ecol..

[36] Bailleul F, Pinaud D, Hindell M, Charrassin JB, Guinet C (2008). Assessment of scale-dependent foraging behaviour in southern elephant seals incorporating the vertical dimension: A development of the First Passage Time method. J. Animal Ecol..

[37] Heerah K, Hindell M, Guinet C, Charrassin J-B (2014). A new method to quantify within dive foraging behaviour in marine predators. PLoS One.

[38] Chelton DB, Schlax MG, Samelson RM (2011). Global observations of nonlinear mesoscale eddies. Prog. Oceanogr.

[39] Lapeyre G, Klein P (2006). Impact of the small-scale elongated filaments on the oceanic vertical pump. J. marine research.

[40] Rosso I (2014). Vertical transport in the ocean due to sub-mesoscale structures: Impacts in the kerguelen region. Ocean Model..

[41] Thompson AF (2016). Open-ocean submesoscale motions: A full seasonal cycle of mixed layer instabilities from gliders. J. Phys. Oceanogr..

[42] du Plessis M, Swart S, Ansorge I, Mahadevan A (2017). Submesoscale processes promote seasonal restratification in the subantarctic ocean. J. Geophys. Res. Ocean..

[43] Viglione, G. A., Thompson, A. F., Flexas, M. M., Sprintall, J. &Swart, S. Abrupt transitions in submesoscale structure in southern drake passage: Glider observations and model results. *J. Phys. Oceanogr*. (2018).

[44] Biggs DC (1992). Nutrients, plankton, and productivity in a warm-core ring in the western gulf of mexico. J. Geophys. Res. Ocean..

[45] Riandey V, Champalbert G, Carlotti F, Taupier-Letage I, Thibault-Botha D (2005). Zooplankton distribution related to the hydrodynamic features in the algerian basin (western mediterranean sea) in summer 1997. Deep Sea Res. Part I: Oceanogr. Res. Pap..

[46] Landry MR, Decima M, Simmons MP, Hannides CC, Daniels E (2008). Mesozooplankton biomass and grazing responses to cyclone opal, a subtropical mesoscale eddy. Deep Sea Res. Part II: Topical Studies in Oceanography.

[47] Le Bras Y, Jouma’a J, Picard B, Guinet C (2016). How Elephant Seals (Mirounga leonina) Adjust Their Fine Scale Horizontal Movement and Diving Behaviour in Relation to Prey Encounter Rate. Plos One.

[48] Guinet C (2014). Southern elephant seal foraging success in relation to temperature and light conditions: Insight into prey distribution. Mar. Ecol. Prog. Ser..

[49] Field IC, Bradshaw CJA, Burton HR, Sumner MD, Hindell MA (2005). Resource partitioning through oceanic segregation of foraging juvenile southern elephant seals (Mirounga leonina). Oecologia.

[50] Bradshaw CJ, Hindell MA, Sumner MD, Michael KJ (2004). Loyalty pays: potential life history consequences of fidelity to marine foraging regions by southern elephant seals. Animal Behaviour.

[51] Rodrguez JP (2017). Big data analyses reveal patterns and drivers of the movements of southern elephant seals. Sci. reports.

[52] Authier, M., Bentaleb, I., Ponchon, A., Martin, C. & Guinet, C. Foraging fidelity as a recipe for a long life: Foraging strategy and longevity in male southern elephant seals. *PLoS One* **7** (2012). 10.1371/journal.pone.0032026 PMC3323586 22505993

[53] Arthur B (2015). Return customers: Foraging site fidelity and the effect of environmental variability in wide-ranging antarctic fur seals. PloS one.

[54] Robinson PW (2012). Foraging behavior and success of a mesopelagic predator in the northeast pacific ocean: insights from a data-rich species, the northern elephant seal. PloS one.

[55] Callies J, Ferrari R, Klymak JM, Gula J (2015). Seasonality in submesoscale turbulence. Nat. communications.

[56] Vallis, G. K. *Atmospheric and oceanic fluid dynamics* (Cambridge University Press, 2017).

[57] Siegelman, L. *et al*. Correction and accuracy of high-and low-resolution ctd data from animal-borne instruments. *Journal of Atmospheric and Oceanic Technology* (2019).

[58] Intergovernmental-Oceanographic-Commission *et al*. The international thermodynamic equation of seawater–2010: calculation and use of thermodynamic properties includes corrections up to 31st october 2015. *UNESCO* (2010).

[59] Schneider N (2000). A decadal spiciness mode in the tropics. Geophys. Res. Lett..

[60] Kilpatrick T, Schneider N, Di Lorenzo E (2011). Generation of low-frequency spiciness variability in the thermocline. J. Phys. Oceanogr..

[61] de Boyer Montégut, C., Madec, G., Fischer, A. S., Lazar, A. & Iudicone, D. Mixed layer depth over the global ocean: An examination of profile data and a profile-based climatology. *J. Geophys. Res. Ocean*. **109** (2004).

[62] Kareiva P, Odell G (1987). Swarms of predators exhibit “preytaxis” if individual predators use area-restricted search. The Am. Nat..

[63] Bailleul F, Lesage V, Hammill MO (2010). Spherical First Passage Time: A tool to investigate area-restricted search in three-dimensional movements. Ecol. Model..

[64] Gallon S (2013). Identifying foraging events in deep diving southern elephant seals, Mirounga leonina, using acceleration data loggers. Deep-Sea Research Part II: Topical Studies in Oceanography.

[65] Thums M, Bradshaw CJ, Hindell MA (2011). *In situ* measures of foraging success and prey encounter reveal marine habitat-dependent search strategies. Ecol..

[66] Fauchald P, Tveraa T (2003). Using first-passage time in the analysis of area-restricted search and habitat selection. Ecol.

[67] Wood, S. N. *Generalized additive models: an introduction with R* (Chapman and Hall/CRC, 2006).

[68] Zuur, A., Ieno, E., Walker, N., Saveliev, A. & Smith, G. Mixed effects models and extensions in ecology with r. gail m, krickeberg k, samet jm, tsiatis a, wong w, editors (New York, NY, Spring Science and Business Media, 2009).

[69] Wood, S. *Generalized Additive Models: An Introduction with R* 2 edn (Chapman and Hall/CRC, 2017).

[70] Kim YS, Orsi AH (2014). On the variability of antarctic circumpolar current fronts inferred from 1992–2011 altimetry. J. Phys. Oceanogr..

