# Supplementary material for: Submesoscale ocean fronts act as biological hotspot for southern elephant seal
Source: Sci Rep. 2019 Apr 3;9:5588. doi: 10.1038/s41598-019-42117-w (PMC6447572; doi:10.1038/s41598-019-42117-w)
Supplement: Supplementary file 1 — Supplementary Information [file 41598_2019_42117_MOESM1_ESM.pdf]

Supplementary Information

Submesoscale ocean fronts act as biological hotspot for southern elephant seal

Lia Siegelman[1,2,3*], Malcolm O'Toole[4], Mar Flexas[2], Pascal Rivière[1], Patrice Klein[2,3]

1. Univ Brest, CNRS, IRD, Ifremer, LEMAR, Plouzané, France
2. California Institute of Technology, Pasadena, CA, USA
3. Jet Propulsion Laboratory, California Institute of Technology, Pasadena, CA, USA
4. UWA Oceans Institute, Indian Ocean Marine Research Centre, University of Western Australia, Crawley, WA 6009, Australia

*Corresponding author : lsiegelman@caltech.edu

Table S1 Results of modelling the seal's foraging effort (magnitude of FPHT) as a smooth function of key physical parameters

	model type	spline type	edf of smooth	p-value	adjusted r2
Strain	GAM	thin plate	8.665	<2e-16	0.565
Lateral gradient of buoyancy at 15 m	GAM	thin plate	2.567	<2e-16	0.055
Lateral gradient of spiciness at 150 m	GAM	thin plate	7.017	<2e-16	0.0825
Mixed layer depth	GAM	thin plate	7.103	<2e-16	0.0538

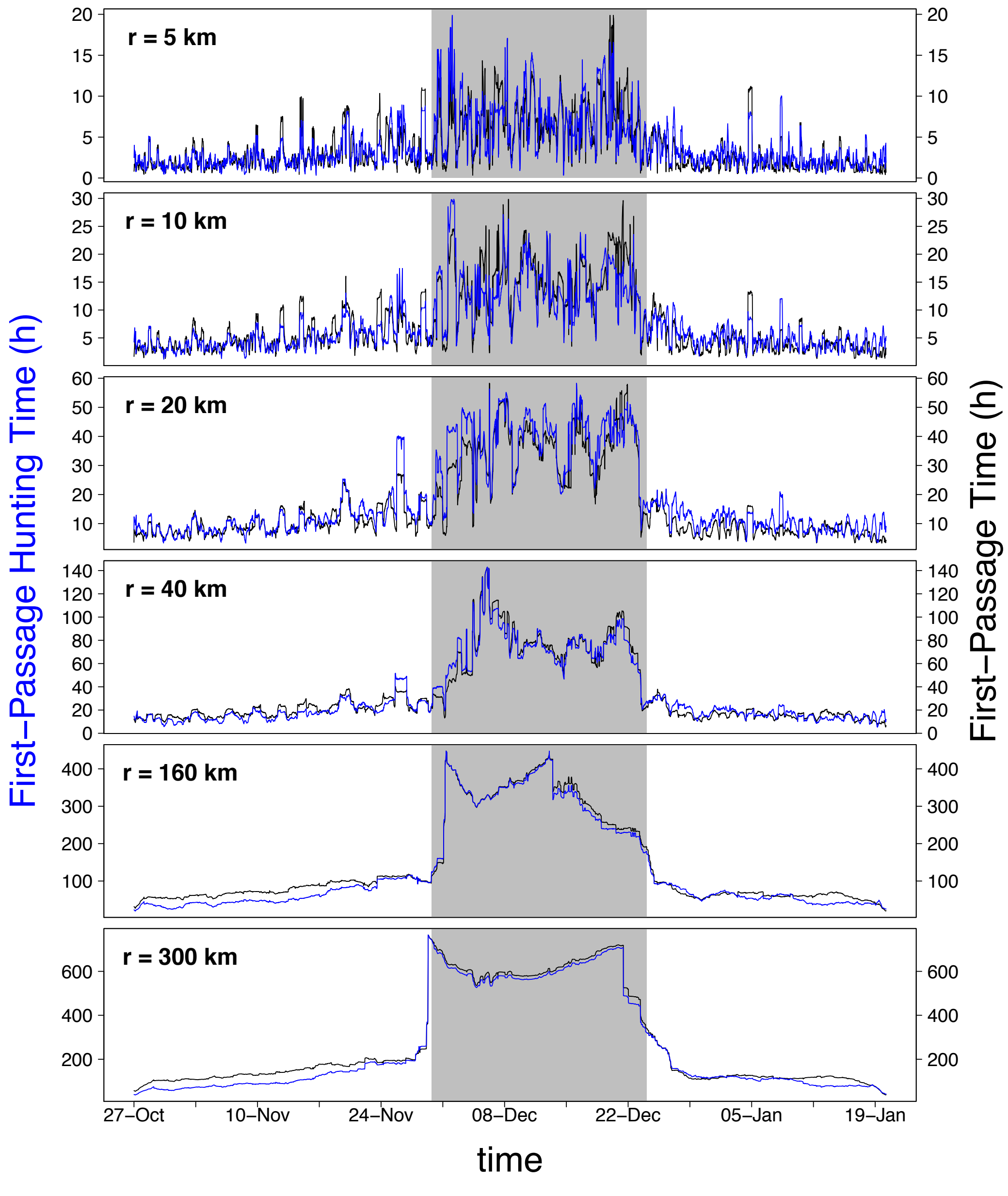

Figure S1. Comparision of First-Passage Hunting Time (FPHT, blue) and First-Passage Time (FPT, black) for different radii (r). Both metrics are similar across scales (or radius r), but also show small differences. On each subplot, the standing meander area is identified by the grey rectangle. This region consistently features the highest FPHT and FPT values.

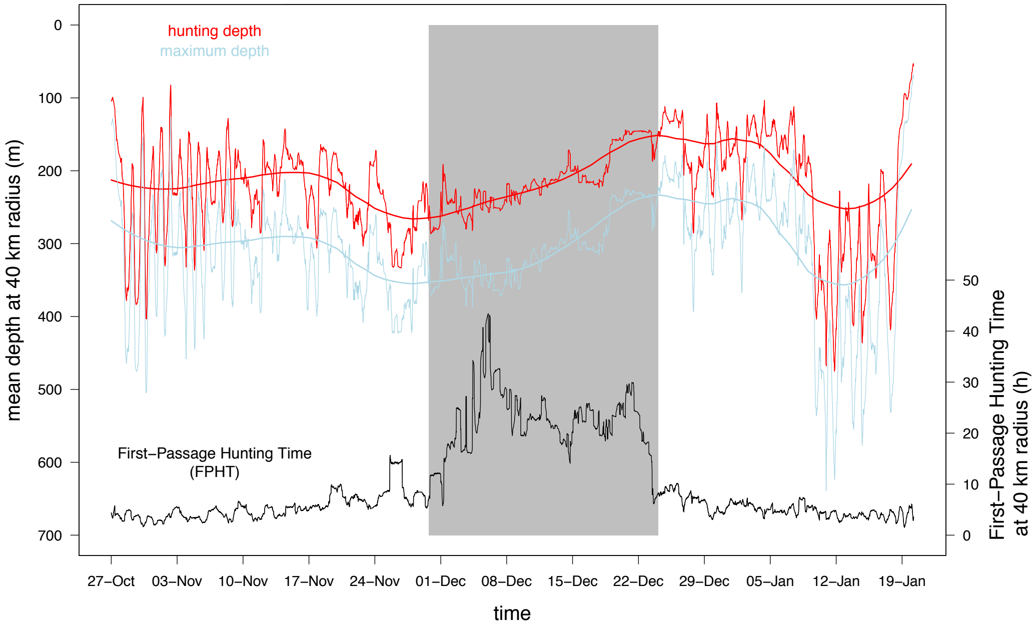

Figure S2. Hunting depth (red) and maximum dive depth (blue) averaged over a 40 km radius. First-passage hunting time (black) and the standing meander area (shaded box) are included for comparison : hunting depth and maximum dive depth variances decreases in the standing meander. Incidentally, dives are progressively shallower as the seal transits north to south, which is consistent with changes in prey vertical accessibility observed by Guinet *et al*. (2014)[45].

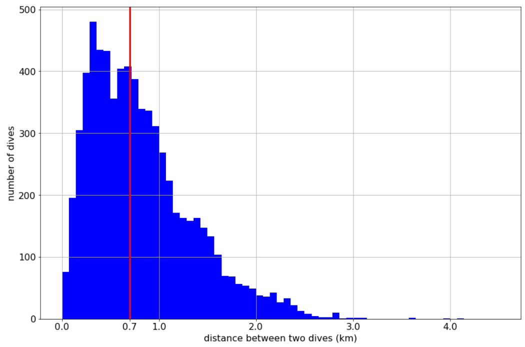

Figure S3. Histogram of the distance between two dives with the median distance of 700 m in red.
